# Triphenylene discotic liquid crystal trimers synthesized by Co_2_(CO)_8_-catalyzed terminal alkyne [2 + 2 + 2] cycloaddition

**DOI:** 10.3762/bjoc.9.321

**Published:** 2013-12-11

**Authors:** Bin Han, Ping Hu, Bi-Qin Wang, Carl Redshaw, Ke-Qing Zhao

**Affiliations:** 1 College of Chemistry and Materials Science, Sichuan Normal University, Chengdu 610066, China; 2 Department of Chemistry, University of Hull, Hull, HU6 7RX, UK

**Keywords:** Co_2_(CO)_8_ catalyzed cycloaddition, columnar mesophase, discotic liquid crystal, triphenylene, oligomer

## Abstract

The synthesis of star-shaped discotic liquid crystal trimers using Co_2_(CO)_8_-catalyzed terminal alkyne [2 + 2 + 2] cycloaddition reaction is reported. The trimers consist of three triphenylene discotic units linked to a central 1,2,4-trisubstituted benzene ring via flexible spacers. The trimers were synthesized in the yields up to 70% by mixing the monomers with 10 mol % of Co_2_(CO)_8_ as the catalyst in refluxing 1,4-dioxane. The liquid crystalline properties were investigated by using polarizing optical microscopy (POM), differential scanning calorimetry (DSC) and X-ray diffraction (XRD). Trimer **4** with an ester connecting group and a longer spacer exhibited a rectangular columnar mesophase, while **5b** and **5c** possessing an ether linkage and a shorter spacer display a hexagonal columnar mesophase. The connecting functional group and the length of the flexible spacer between the central benzene ring and the triphenylene units have pivotal influence on the mesomorphism.

## Introduction

Discotic liquid crystals (DLCs) with nematic phase have been commercially utilized in the liquid crystal display industry as optical compensating films for widening the view angles [1–2]. More interestingly, DLCs can self-organize into columnar mesophases with a high degree of order, and show fast unidirectional charge migration properties, and have been studied as soft organic semiconductors [3–12]. Solution-processed and ink-jet printing organic electronic devices based on liquid crystalline semiconductors are low-cost and thus are especially attractive to industry. Until now, DLCs have been explored as active materials applied in organic light-emitting diodes (OLEDs) [13–15], organic field-effect transistors (OFETs) [5–8,16–17], and organic photovoltaic solar cells (OPVs) [18–20].

The reported DLC oligomers [21–50] are limited compared with the low-molar-mass DLCs and polymeric DLC materials, due to the construction methods for the oligomers. However, DLC oligomers usually possess wider mesophase ranges than the monomers as the crystallization was prevented, due to the size of molecules is enlarged and molecular symmetry is lowered. However DLC oligomers exhibit higher charged carrier mobility than DLC polymers, the most important parameter in determining the device performance, as the discotic unites can still self-assemble to higher order through the π–π interaction. In addition, DLC oligomers similar to polymers can be processed on flexible substrates by spin-casting, screen printing, doctor-blading, ink-jet printing and roll-to-roll processing, so that these cost-effective deposition methods can be used to manufacture electronic devices. Therefore, the exploration of efficient synthetic methods for the DLC oligomers and studying the properties of them are fundamental.

The construction methods of DLC oligomers can be divided into conventional organic reactions and transition metal-catalyzed synthetic methods. The use of transition metal-mediated reactions for constructing new organic functional materials is more efficient and therefore attractive. Our group has embarked upon a program to use new organic synthetic methods to prepare novel types of DLC materials and to study the relationship between their molecular structures and the mesomorphic properties [46–50]. We have reported that Cu(I)-catalyzed alkyne–azide click reactions are emerging as an efficient method for the synthesis of discotic oligomers [46–50].

The transition metal-catalyzed [2 + 2 + 2] cycloaddition of three alkynes for the synthesis of polysubstituted benzene derivatives was studied due to its high efficiency and atomic efficiency [51]. The use of Co_2_(CO)_8_ as a catalyst has been extensively applied to the synthesis of hexabenzocoronene DLCs through diarylethyne cyclotrimerization [51].

**Scheme 1:**
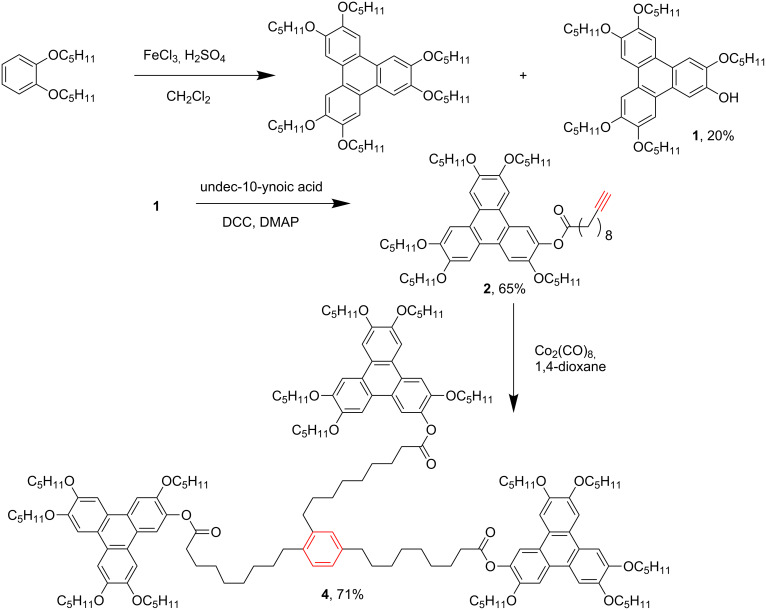
Synthesis of star-shaped triphenylene discotic liquid crystalline trimer **4**.

**Scheme 2:**
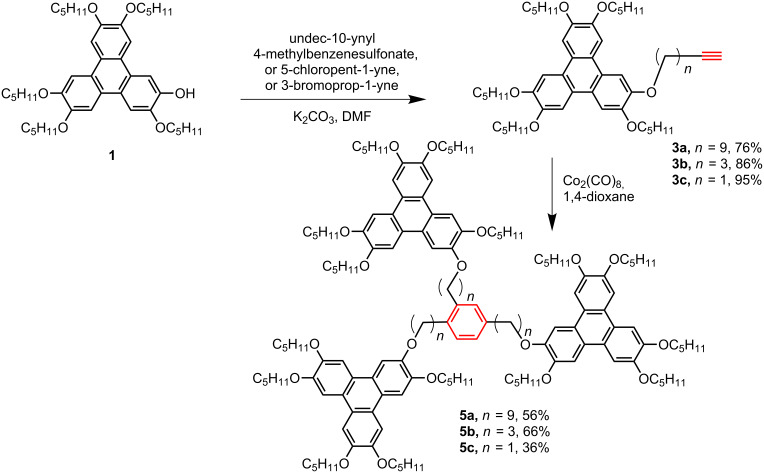
Synthesis of star-shaped triphenylene discotic liquid crystalline trimers **5a–c**.

In this paper, we report four star-shaped DLC trimers with triphenylene discotic units by using a Co_2_(CO)_8_-catalyzed terminal alkynes [2 + 2 + 2] cycloaddition, and the trimers exhibit ordered rectangular (Col_ro_) and hexagonal columnar mesophases (Col_ho_). Furthermore, the structure-mesomorphic property relationship is discussed. The synthetic route is shown in Scheme 1 and Scheme 2.

## Results and Discussion

### Synthesis and characterization

We synthesized the key intermediate **1**, 2-hydroxy-3,6,7,10,11-pentakis(pentyloxy)triphenylene, according to a simplified one-pot method [52]. The undec-10-yn-1-yl *p*-toluenesulfonate was prepared by LiAlH_4_ reduction of the acid and tosylation of the alcohol [53–54]. Then monomers **2** and **3a–c** were synthesized by the direct esterification or etherification reaction between phenol **1** and 10-undecynoic acid, undec-10-yn-1-yl *p*-toluenesulfonate, 5-chloro-1-pentyne, propargyl bromide, respectively.

The star-shaped DLC trimers with triphenylene discogens, **4** and **5a–c**, were synthesized in yields of 36–71% by the self-trimerization of monomer **2** or **3a–c** catalyzed by using 10 mol % of Co_2_(CO)_8_ in refluxing 1,4-dioxane. Trimer **4**, **5a** and **5b** were prepared in moderate yields, and the obvious lower synthetic yield of **5c** might be caused by its shorter spacer and bigger steric hindrance. Considering the size of the trimers, we were satisfied with the preliminary synthetic yields, and did not further optimize the reaction conditions.

Two isomers were obtained in the trimerization of mono-substituted alkynes, R-C≡CH: 1,2,4- and 1,3,5-trialkylbenzene. For the DLC trimers, the isomers could not be separated by thin-layer chromatography and column chromatography, and even high performance liquid chromatography (HPLC). However, the benzenes with three substituents, a 1,2,4- or 1,3,5-trisubstituted pattern, can be characterized by ^1^H NMR spectroscopy [55–56]. According to this method, we find that the ^1^H NMR peak of the 1,3,5-trisubstituted benzene isomer **4** appears at 6.83 ppm, for **5a** at 6.81 ppm, and 7.03 ppm for **5b**. There was no signal for the 1,3,5-trisubstituted isomer for **5c**. The ^1^H NMR peak area integration results showed that for **4**, **5a** and **5b**, the 1,2,4-trisubstituted benzenes were present in more than 95% and 1,3,5-trisubstituted benzene isomers were less than 5%. For **5c**, the symmetric isomer of the 1,3,5-trisubstituted benzene was not detected, and the yield of the 1,2,4-trisubstituted isomer was almost quantitative. Therefore, we came to the conclusion that this synthetic method and the following purification procedures supplied the 1,2,4-trisubstituted benzene-cored DLC oligomers.

### Mesomorphism

#### POM and DSC

**Figure 1:**
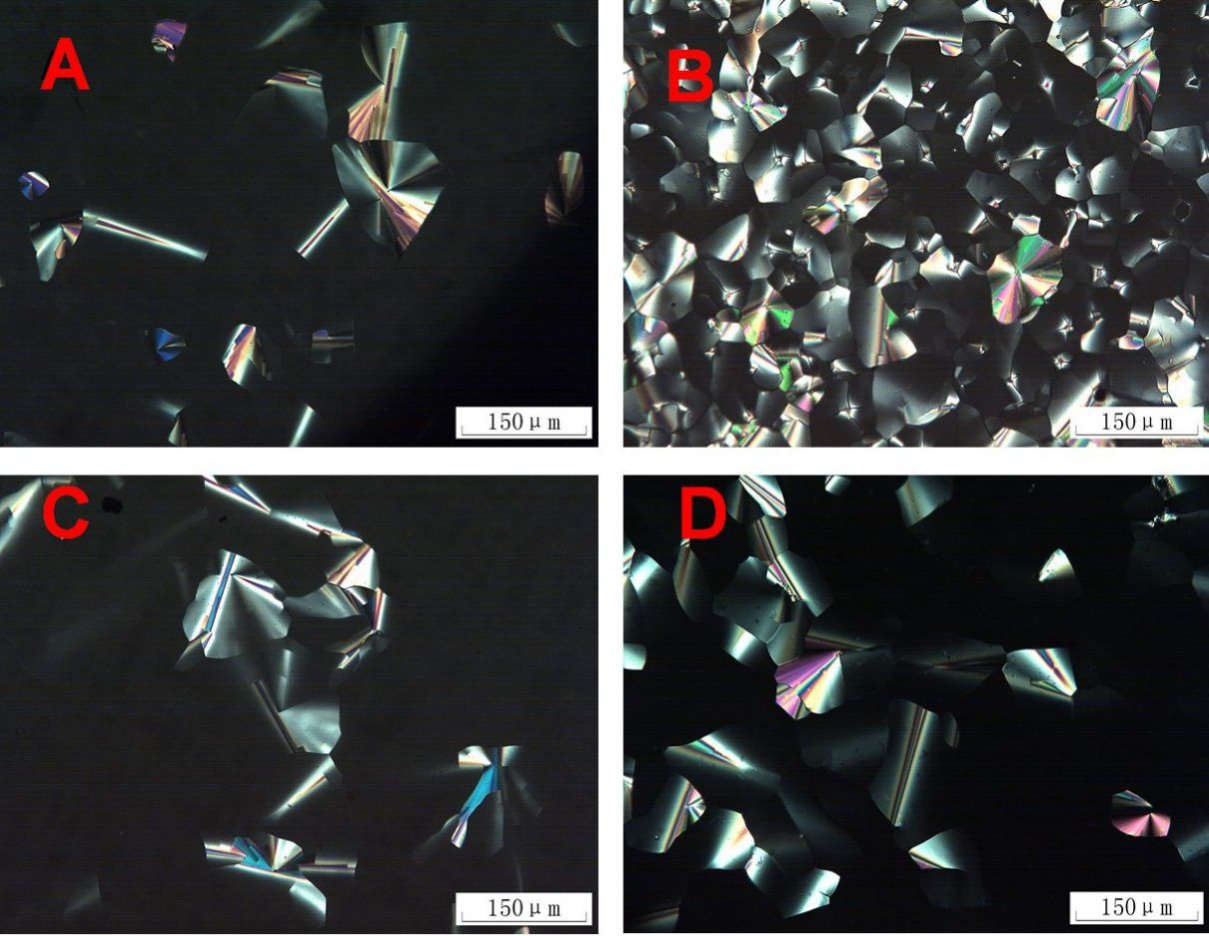
Optical photomicrographs of the triphenylene DLC monomers. (A) **2** at 40 °C; (B) **3a** at 45 °C; (C) **3b** at 62 °C; (D) **3c** at 67 °C.

**Figure 2:**
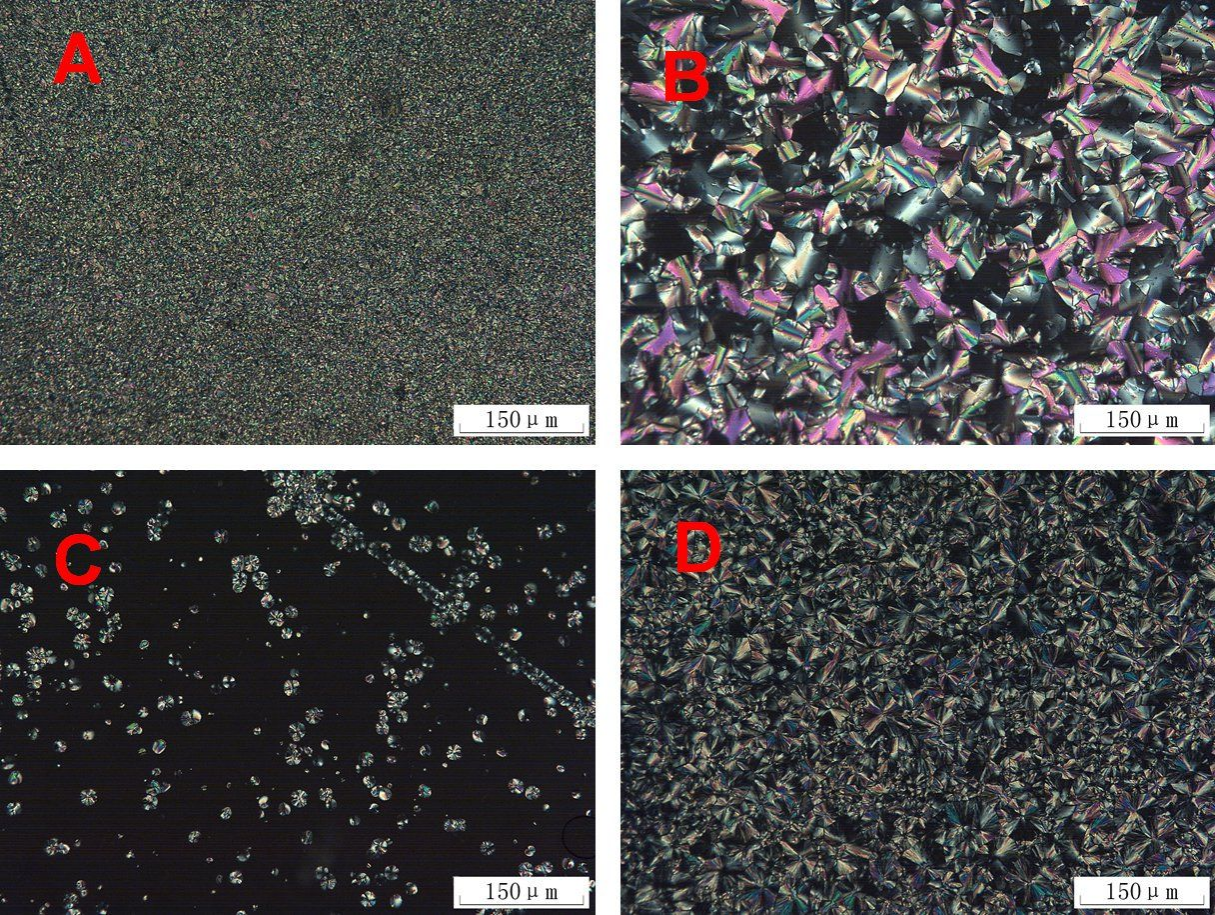
Optical photomicrographs of the triphenylene DLC trimers. (A) **4** at 70 °C; (B) **5b** at 85 °C; (C) **5c** at 100 °C; (D) **5c** at 75 °C.

**Figure 3:**
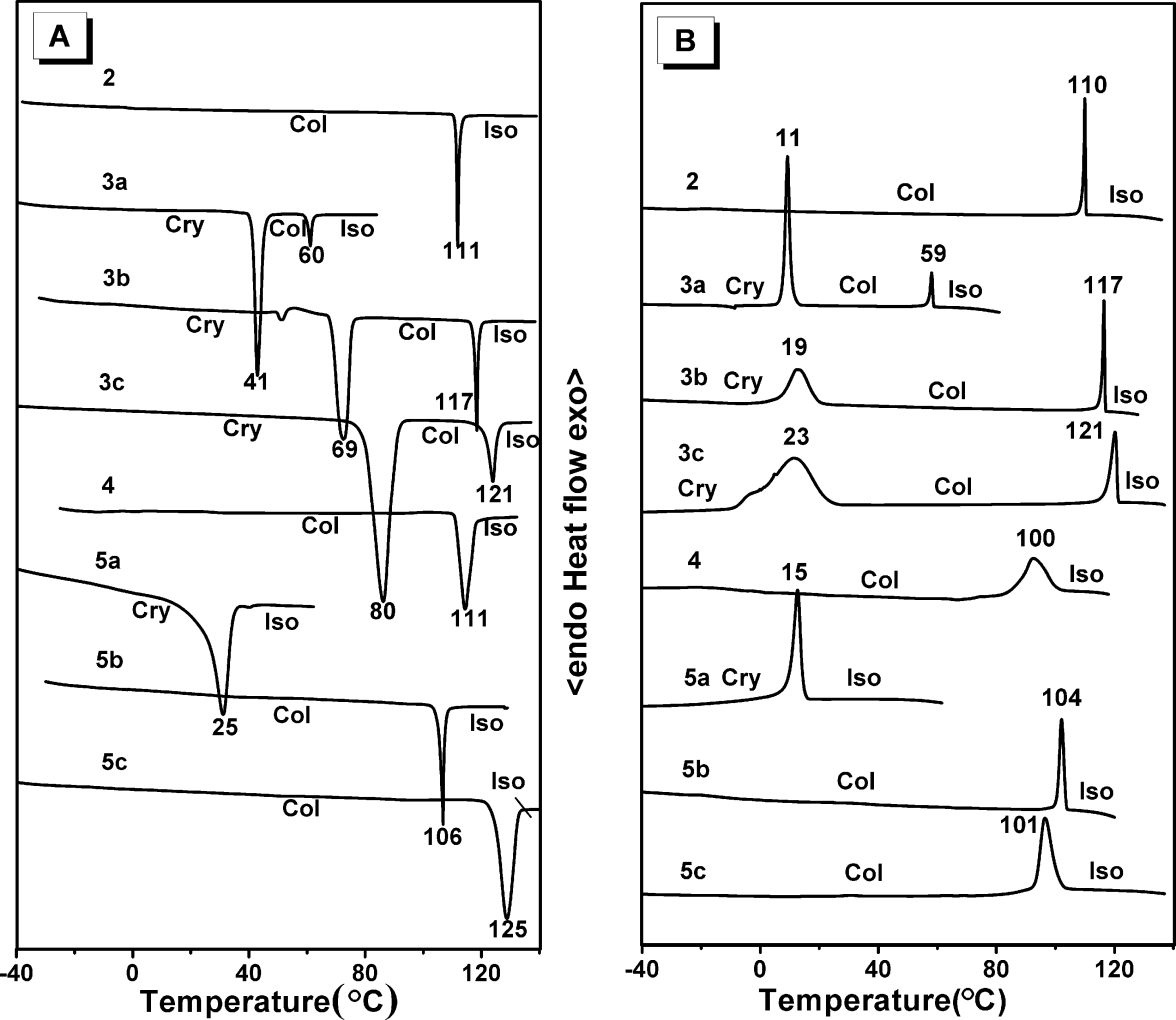
The DSC traces of the triphenylene DLC monomers and trimers. (A) 2nd heating traces; (B) 1st cooling traces. Scanning rate 10 K/min.

Initially, we studied the mesomorphic properties of the monomers and trimers by using polarizing optical microscopy (POM) and differential scanning calorimetry (DSC). The POM results of the monomers and trimers are summarized in Figure 1 and Figure 2, respectively. The DSC traces are shown in Figure 3 and the phase transition data are summarized in Table 1.

**Table 1:** Thermotropic phase-transition behavior of the triphenylene DLC monomers and trimers. (Heating and cooling rate of 10 K/min.)^a^

Compd.	2nd heating	1st cooling

	Transition temperature (°C) and enthalpy change (Δ*H*, kJ/mol)	Transition temperature (°C) and enthalpy change (Δ*H*, kJ/mol)

TP(OC_5_H_11_)_6_	69 Col_ho_ 122	
**2**^b^	Col 111 (9.8) Iso	Iso 110 (9.8) Col
**3a**	Cr 41 (47.8) Col 60 (4.8) Iso	Iso 59 (5.1) Col 11 (43.0) Cr
**3b**^b^	Cr 69 (44.2) Col 117 (10.3) Iso	Iso 117 (10.4) Col 19 (26.5) Cr
**3c**	Cr 80 (67.5) Col 121 (11.4) Iso	Iso 121 (11.1) Col 23 (46.7) Cr
**4**	Col 111 (9.5) Iso	Iso 100 (4.7) Col
**5a**	Cr 25 (14.3) Iso	Iso 15 (14.6) Cr
**5b**	Col 106 (10.9) Iso	Iso 104 (10.5) Col
**5c**	Col 125 (19.1) Iso	Iso 101 (10.4) Col

^a^Cr, crystal state; Col, columnar phase; Iso, isotropic liquid. ^b^Monomer **2** [52] and **3b** [57] were reported and the mesomorphism is comparable.

For the mesomorphism of the functionalized triphenylene monomers, compound **2** [52] and **3b** [57] have been reported, **3a** and **3c** are new. They all display typical optical textures with homeotropic alignment behavior of the hexagonal columnar (Col_h_) mesophase (Figure 1). The monomers display different phase-transition temperatures related to the connecting functional group and the length of the chain. Compound **2** displayed a Col phase at room temperature with a clearing point of 111 °C (for the heating run), and did not crystallize even when cooled to −50 °C. Monomer **3a** exhibited a narrow columnar mesophase range between 41 °C to 60 °C for heating, and between 59 °C to 11 °C for cooling. Comparing the phase-transition temperatures of **2** and **3a**, it was found that the ester connecting group has the effect of lowering the melting point and rising the clearing point. Compared with the symmetric discogen of 2,3,6,7,10,11-hexakis(pentyloxy)triphenylene (C5OTP) which possesses a Col phase between 69–122 °C [58], monomer **2** displays a lower melting point and **3a** exhibits both lowered melting and clearing points. The ester connecting group of **2** increases its dipole–dipole interactions in the intermolecular columnar stacking and stabilizes the Col mesophase. The results here are in agreement with the earlier works of Wendorff, Ringsdorf and Spiess [59–64].

Both **3b** and **3c** having slightly lowered molecular symmetry compared to the parent compound C5OTP, exhibited a Col mesophase between 69–117 °C and 80–121 °C, respectively.

Figure 2 shows the POM results for the trimers. Trimer **4** self-assembled into small-sized domains which were independent of the temperature and the cooling rate, and displayed a clearing point at 110 °C on heating, and on the cooling run the Col phase appeared at 100 °C. The crystallization did not occur for **4** even when it was cooled to −50 °C at the rate of 10 K/min.

Trimer **5a** did not show mesomorphism as indicated both from the results of the POM and the DSC. The DSC curves of **5a** showed only one phase transition peak at 15 °C on the first cooling run and at 25 °C on the second heating run. From the POM observations, we noted that the peak represents the crystal to isotropic liquid transition (Cr→Iso) and the reversed Iso→Cr transition.

Both trimer **5b** and **5c** exhibited a similar mesophase behavior to trimer **4**: There is only one phase transition peak on the first cooling process and the second heating run. For **5b**, the Col→Iso transition occurred at 106 °C on heating, and Iso→Col appeared at 104 °C on cooling. For **5c**, the Col→Iso transition occurred at 125 °C on heating, and the reversed transition at 101 °C on cooling. No crystallization was observed for either **5b** or **5c**.

### XRD results

**Figure 4:**
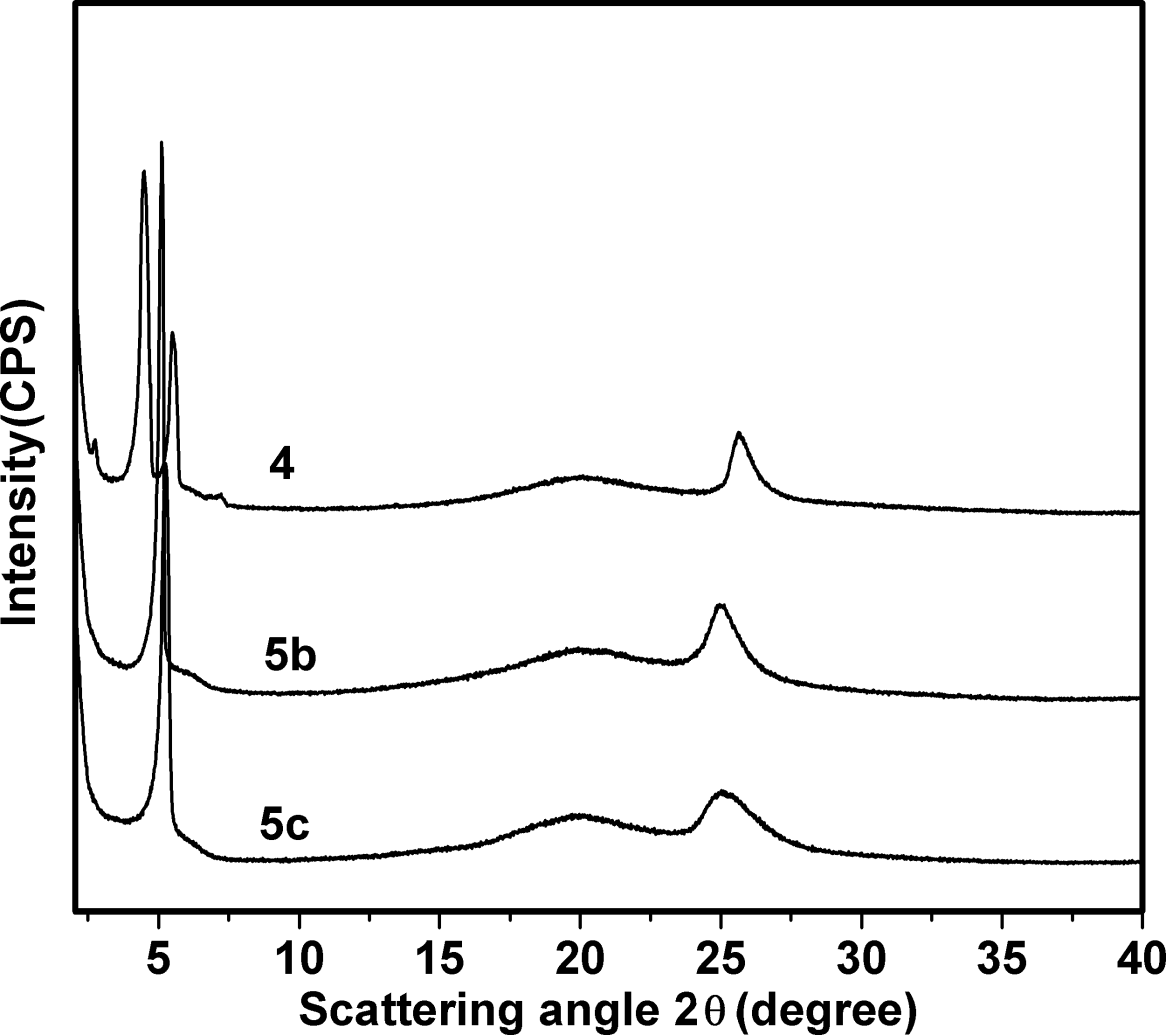
Powder X-ray diffraction patterns of the DLC trimmers **4**, **5b** and **5c** at room temperature.

Figure 4 depicts the X-ray diffraction patterns of **4**, **5b** and **5c** at room temperature cooling from the isotropic liquid. Both **5b** and **5c** show a strong diffraction peak in the small-angle region (2θ = 3.8° or 4.5°), a broad halo peak of the alkyl chain at ca. 18°, and a core–core distance peak at 3.5–3.7 Å. Considering these XRD results together with their homeotropic alignment behavior displayed by the POM results shown in Figure 2C and Figure 2D, we assigned the mesophase of **5b** and **5c** as the Col_ho_ phase.

**Figure 5:**
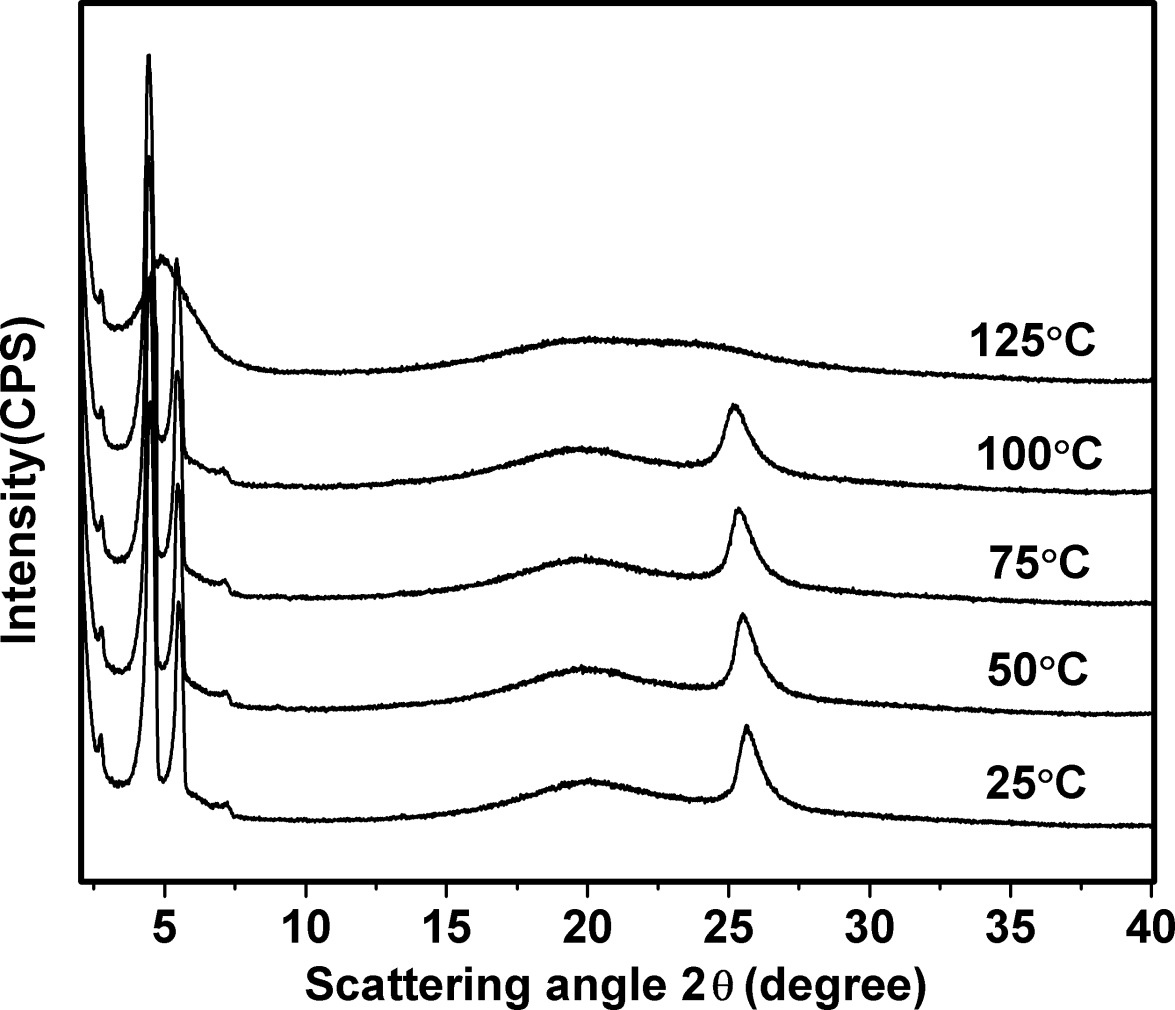
X-ray diffraction profiles of **4** at different temperatures.

However, **4** exhibited a different XRD pattern from that of **5b** and **5c**: two strong diffraction peaks in the small-angle region (2θ = 3.7° and 4.7°) with a d value of 23.78 Å and 18.80 Å, respectively. We assigned the mesophase of **4** as the rectangular columnar phase (Col_ro_), which was further confirmed by the temperature-dependent XRD results (Figure 5), and the non-homeotropic alignment behavior of the POM texture (Figure 2A). The lattice parameters of the discotic Col mesophase of the trimers are summarized in Table 2. Trimer **4** displayed a smaller intracolumnar core–core distance of 3.57Å than that of **5b** and **5c**, due to the stronger π–π interactions between the triphenylene discogens with mono-ester group. The columnar parameter values of the trimers decreased with the spacer length shortened. So we deduced that the benzene cores were among the alkyl chains in the columnar stacking of the trimers.

**Table 2:** Columnar mesophase parameters of the DLC trimers.

Compd.	*hkl*	d*_hkl_* (Å)	lattice parameters (Å)

**4**	200	23.78	*a* = 47.56 (Col_ro_)
(25 °C)	110	18.80	*b* = 20.47
	alkyl halo	4.70	
	001	3.57	
**5b**	100	22.97	26.52 (Col_ho_)
(25 °C)	alkyl halo	4.87	
	001	3.75	
**5c**	100	19.70	22.75 (Col_ho_)
(25 °C)	alkyl halo	4.87	
	001	3.65	

Further considering the relationship between the molecular structures of the trimers and the mesomorphism, we noted first the mesophase disappearance of **5a**, which was synthesized by trimerization of liquid crystalline **3a**. However, trimer **4** has displayed a stable Col_ro_ mesophase while **5a** did not. Both **4** and **5a** possess the same length spacer, the ester-linker of **4** has a stronger polarity than the ether-linker of **5a**. The isotropic point of **4** (111 °C) is higher than that of **5a** (25 °C). More importantly, **4** is a room temperature liquid crystal and **5a** has not shown a mesophase. Compared with **5a**, both **5b** and **5c** have shorter spacers and have displayed a stable Col_ho_ mesophase over a wide temperature range.

Our study has demonstrated that for the star-shaped DLC trimers, both the length of spacer and the connecting linker group to the discotic units play a crucial role in the formation and stabilization of the discotic columnar mesophase.

## Conclusion

The synthesis and mesomorphism of two new mono-functionalized triphenylene discotic monomers and four discotic trimers is reported. The trimers have been successfully synthesized for the first time by using a Co_2_(CO)_8_-catalyzed terminal alkyne [2 **+** 2 **+** 2] cycloaddition reaction in moderate yields. Three of the four 1,2,4-trisubstituted benzene-cored discotic trimers have shown stable Col_ho_ and Col_ro_ mesophases and wide mesophase ranges including room temperature. The connecting linker group to the triphenylene and the spacer length to the central benzene core have important impacts on the supramolecular packing and phase-transition temperature. We anticipate that this Co_2_(CO)_8_-catalyzed synthetic method can be used for the synthesis of liquid crystalline polymer networks.

## Experimental

Instruments and conditions: See Supporting Information File 1.

### Chemical reagents and starting materials

10-undecynoic acid, 5-chloro-1-pentyne, propargyl bromide and Co_2_(CO)_8_ were purchased from Alfa Aesar (Tianjin, China). All the other reagents and solvents were commercial products and used without further purification unless otherwise noted. 10-Undecyn-1-yl *p*-toluenesulfonate [53–54], 2-hydroxy-3,6,7,10,11-pentakis(pentyloxy)triphenylene [52] were synthesized as described in the reported methods.

**Monomer 2, C_18_H_6_(OC_5_H_11_)_5_(OOCC_8_H_16_-C≡CH):** Monomer 2 was prepared similar to the reported method [52]. The mixture of 10-undecynoic acid (218 mg, 1.2 mmol), dicyclohexylcarbodiimide (DCC, 247 mg, 1.2 mmol), 4-*N*,*N*-dimethylaminopyridine (DMAP, 67 mg), and 2-hydroxy-3,6,7,10,11-pentakis(pentyloxy)triphenylene (**1**, 674 mg, 1.0 mmol) in CH_2_Cl_2_ (20 mL) was stirred at room temperature for 6 h under a nitrogen atmosphere. After the reaction was finished, water was added to the mixture and then extracted with CH_2_Cl_2_. The extract was dried with MgSO_4_ and the solvent was distilled off using a rotary evaporator. The crude product was purified by column chromatography on silica gel eluted with CH_2_Cl_2_/hexane (3:2 in volume). The product was re-crystallized from ethanol affording a white solid (543 mg, 0.65 mmol, 65%). ^1^H NMR (CDCl_3_, TMS, 400 MHz) δ 8.06 (s, 1H, ArH), 7.85–7.77 (m, 5H, ArH), 4.26–4.19 (m, 10H, CH_2_), 2.67 (t, *J* = 7.6 Hz, 2H, CH_2_), 2.20 (td, *J* = 7.2 Hz, *J* = 2.8 Hz, 2H, CH_2_), 1.96–1.87 (m, 13H, CH_2_, C≡CH), 1.57–1.39 (m, 30H, CH_2_), 0.99–0.97 (t, *J* = 7.2 Hz, 15H, CH_3_).

**Monomer 3a, C_18_H_6_(OC_5_H_11_)_5_(OC_9_H_18_-C≡CH):** The mixture of 10-undecyn-1-yl *p*-toluenesulfonate (387 mg, 1.2 mmol), K_2_CO_3_ (345 mg, 2.5 mmol), and 2-hydroxy-3,6,7,10,11-pentakis(pentyloxy)triphenylene **(1**, 74 mg, 1.0 mmol) in DMF (15 mL) was stirred at 80 °C for 24 h under N_2_. The mixture was cooled to room temperature, and 3 M HCl was added drop-wise until the mixture was acidic. The organic phase was extracted with CH_2_Cl_2_ and dried over anhydrous MgSO_4_. The solvent was evaporated under reduced pressure to afford the crude product, which was purified by column chromatography on silica gel eluted with CH_2_Cl_2_/hexane. The product was re-crystallized from ethanol to afford a light yellow solid (626 mg, 0.76 mmol, 76%). ^1^H NMR (CDCl_3_, TMS, 400 M) δ 7.84 (s, 6H, ArH), 4.23 (t, *J* = 6.4 Hz, 12H, CH_2_), 2.21–2.17 (m, 2H, CH_2_) 1.99–1.92 (m, 13H, CH_2_, C≡CH), 1.60–1.50 (m, 14H, CH_2_), 1.49–1.36 (m, 18H, CH_2_), 0.99–0.96 (m, 15H, CH_3_); ^13^C NMR (CDCl_3_, 100 MHz) δ 148.9, 123.6, 107.2, 84.7, 69.6, 68.1, 29.5, 29.1, 28.8, 28.5, 28.4, 26.2, 22.6, 18.4, 14.2; anal. calcd for C_54_H_80_O_6_: C, 78.60; H, 9.77; found: C, 78.36; H, 9.68.

**Monomer 3b, C_18_H_6_(OC_5_H_11_)_5_(OC_3_H_6_-C≡CH):** Monomer **3b** was synthesized in a similar way to literature [57]. A mixture of 5-chloro-1-pentyne (230 mg, 2.25 mmol), K_2_CO_3_ (621 mg, 4.50 mmol), and 2-hydroxy-3,6,7,10,11-pentakis(pentyloxy)triphenylene (**1**, 1011 mg, 1.5 mmol) in DMF (20 mL) was stirred at 80 °C for 24 h under N_2_. The crude product was purified through column chromatography and a white solid was obtained (959 mg, 1.3 mmol, 86%). ^1^H NMR (CDCl_3_, TMS, 400 MHz) δ 7.87–7.84 (m, 6H, ArH), 4.34 (t, *J* = 6.0 Hz, 2H, CH_2_), 4.23 (t, *J* = 6.4 Hz, 10H, CH_2_), 2.54 (td, *J* = 7.2 Hz, *J* = 2.8 Hz, 2H, CH_2_), 2.18–2.14 (m, 2H, CH_2_), 2.00 (t, *J* = 2.8 Hz, 1H, C≡CH), 1.99–1.92 (m, 10H, CH_2_), 1.60–1.50 (m, 10H, CH_2_), 1.49–1.41 (m, 10H, CH_2_), 0.98 (t, *J* = 7.2 Hz, 15H, CH_3_); ^13^C NMR (CDCl_3_, 100 MHz), δ 149.0, 148.9, 148.6, 123.8, 123.6, 123.5, 107.6, 107.2, 107.1, 83.7, 69.6, 68.9, 68.0, 29.1, 28.4, 22.6, 15.3, 14.1; anal. calcd for C_48_H_68_O_6_: C, 77.80; H, 9.25; found: C, 77.70; H, 9.33.

**Monomer 3c, C_18_H_6_(OC_5_H_11_)_5_(OCH_2_-C≡CH):** A mixture of propargyl bromide (268 mg, 2.25 mmol), K_2_CO_3_ (621 mg, 4.5 mmol), and 2-hydroxy-3,6,7,10,11-pentakis(pentyloxy)triphenylene (**1**, 1011 mg, 1.5 mmol) in DMF (20 mL) was stirred at 80 °C for 24 h under N_2_. The crude product was purified with column chromatography and a white solid was collected (1013 mg, 1.42 mmol, 95%). ^1^H NMR (CDCl_3_, TMS, 400 MHz) δ 8.08 (s, 1 H, ArH), 7.85 (s, 1H, ArH), 7.83 (s, 4H, ArH), 4.97 (d, *J* = 1.2 Hz, 2H, CH_2_), 4.27–4.22 (m, 10H, CH_2_), 2.58 (t, *J* = 2.4 Hz, 1H, C≡CH), 1.99–1.92 (m, 10H, CH_2_), 1.60–1.53 (m, 10H, CH_2_), 1.50–1.43 (m, 10H, CH_2_), 0.98 (t, *J* = 7.6Hz, 15H, CH_3_); ^13^C NMR (CDCl_3_, 100 MHz) δ 149.2, 149.0, 148.8, 146.7, 124.7, 123.9, 123.5, 123.4, 123.3, 123.2, 109.8, 107.4, 107.2, 107.0, 106.7, 106.5, 78.9, 75.9, 69.7, 69.5, 69.3, 57.8, 29.1, 29.0, 28.4, 28.3, 22.6, 14.1; anal. calcd for C_46_H_64_O_6_: C, 77.49; H, 9.05; found: C, 77.41; H, 9.00.

**Trimer 4:** A mixture of **2** (252 mg, 0.3 mmol) and Co_2_(CO)_8_ (10 mg, 0.03 mmol) in dioxane (20 mL) was stirred under reflux for 24 h under an argon atmosphere, then cooled to room temperature. The organic phase was extracted with CH_2_Cl_2_, dried over MgSO_4_ and the solvent was removed. The crude product was purified by column chromatography on silica gel eluted with CH_2_Cl_2_/hexane (3:1 in volume), and then re-crystallized from ethanol affording a white solid (178 mg, 0.21 mmol, 71%). ^1^H NMR (CDCl_3_, TMS, 600 MHz) δ 8.02–8.00 (m, 3H, ArH), 7.81–7.71 (m, 15H, ArH), 7.06 (d, *J* = 3.6 Hz, 1H, ArH), 6.97 (s, 1H, ArH), 6.95 (d, *J* = 3.9 Hz, 1H, ArH), 6.83 (the 1,3,5-trisubstituted benzene core), 4.23–4.18 (m, 30H, CH_2_), 2.66 (t, *J* = 7.5 Hz, 6H, CH_2_), 2.61–2.55 (m, 6H, CH_2_), 1.97–1.81 (m, 36H, CH_2_), 1.66–1.34 (m, 90H, CH_2_), 0.99–0.95 (m, 45H, CH_3_); ^13^C NMR (CDCl_3_, 100 MHz) δ 172.1, 149.5, 149.3, 149.0, 148.7, 148.6, 140.3, 140.2, 139.6, 137.6, 129.2, 129.0, 127.8, 125.8, 124.5, 123.3, 123.0, 122.9, 116.6, 107.7, 107.0, 106.6, 106.3, 105.8, 69.7, 69.3, 69.1, 68.7, 35.7, 34.2, 32.9, 32.4, 31.7, 31.5, 30.0, 29.9, 29.6, 29.5, 29.4, 29.2, 29.1, 28.4, 28.3, 25.2, 22.6, 14.2; anal. calcd for C_162_H_234_O_21_: C, 77.29; H, 9.37; found: C, 77.61; H, 9.39.

**Trimer 5a:** Trimer **5a** was synthesized by the same method as **4**, which afforded a colorless oily product (186 mg, 0.23 mmol, 56%). ^1^H NMR (CDCl_3_, TMS, 600 MHz) δ 7.82 (s, 18H, ArH), 7.04 (d, *J* = 3.9 Hz, 1H, ArH), 6.95 (s, 1H, ArH), 6.93 (d, *J* = 3.9 Hz, 1H, ArH), 6.81 (the 1,3,5-trisubstituted benzene core), 4.23–4.21 (m, 36H, CH_2_), 2.58–2.52 (m, 6H, CH_2_), 1.97–1.91 (m, 36H, CH_2_), 1.58–1.35 (m, 96H, CH_2_), 0.98–0.95 (m, 45H, CH_3_); ^13^C NMR (CDCl_3_, 100 MHz) δ 149.1, 149.0, 148.9, 148.8, 148,7, 140.3, 140.1, 137.6, 129.2, 128.9, 125.7, 123.7, 123.6, 123.5, 123.4, 107.3, 107.2, 107.1, 107.0, 69.6, 35.7, 32.8, 32.4, 31.7, 31.5, 31.4, 30.0, 29.9, 29.7, 29.6, 29.5, 29.1, 28.4, 26.3, 22.6, 14.1; anal. calcd for C_162_H_240_O_18_: C, 78.60; H, 9.77; found: C, 78.55; H, 9.79.

**Trimer 5b: 5b** was synthesized by the same method as **4**, which afforded a white solid (195 mg, 0.26 mmol, 66%). ^1^H NMR (CDCl_3_, TMS, 600 MHz) δ 7.82–7.73 (m, 18H, ArH), 7.22 (d, *J* = 3.9 Hz, 1H, ArH), 7.18 (s, 1H, ArH), 7.09 (dd, *J* = 3.9 Hz, *J* = 0.9 Hz, 1H, ArH), 7.03 (the 1,3,5-trisubstituted benzene core), 4.28–4.13 (m, 36H, CH_2_), 3.0 (t, *J* = 7.9 Hz, 4H, CH_2_), 2.88 (t, *J* = 8.0 Hz, 2H, CH_2_), 2.28–2.18 (m, 6H, CH_2_), 1.97–1.85 (m, 30H, CH_2_), 1.59–1.35 (m, 60H, CH_2_), 0.99–0.87 (m, 45H, CH_3_); ^13^C NMR (CDCl_3_, 100 MHz) δ 148.9, 148.8, 148.7, 148.6, 139.8, 139.7, 137.2, 129.8, 129.6, 126.4, 123.6, 123.5, 123.4, 107.2, 107.1, 106.9, 106.8, 69.6, 69.5, 69.4, 69.3, 68.7, 68.5, 32.0, 31.9, 31.3, 31.2, 31.1, 29.7, 29.2, 29.0, 28.6, 28.4, 22.6, 14.1; anal. calcd for C_144_H_204_O_18_: C, 77.80; H, 9.25; found: C, 77.83; H, 9.26.

**Trimer 5c:** Trimer **5c** was synthesized by the same method as **4**, which afforded a white solid (117 mg, 0.16 mmol, 36%). ^1^H NMR (CDCl_3_, TMS, 600 MHz) δ 7.95–7.60 (m, 21H, ArH), 5.65 (s, 4H, CH_2_), 5.37 (s, 2H, CH_2_), 4.24–4.12 (m, 22H, CH_2_), 4.07–4.03 (m, 4H, CH_2_), 3.97–3.94 (m, 4H, CH_2_), 1.98–1.86 (m, 22H, CH_2_), 1.80–1.73 (m, 4H, CH_2_), 1.70–1.65 (m, 4H, CH_2_), 1.59–1.37 (m, 44H, CH_2_), 1.36–1.30 (m, 8H, CH_2_), 1.28–1.16 (m, 8H, CH_2_), 1.01–0.94 (m, 27H, CH_3_), 0.93–0.90 (m, 6H, CH_3_), 0.83–0.78 (m, 9H, CH_3_), 0.75 (t, *J* = 7.3 Hz, 3H, CH_3_); ^13^C NMR (CDCl_3_, 100 MHz) δ 149.0, 148.9, 148.8, 137.6, 136.1, 135.4, 129.2, 128.1, 127.2, 124.0, 123.3, 108.7, 107.2, 107.0, 106.9, 106.6, 106.5, 105.9, 71.6, 70.5, 70.2, 69.7, 69.6, 69.5, 69.4, 69.1, 69.0, 29.2, 29.1, 29.0, 28.4, 28.3, 22.6, 14.1; anal. calcd for C_138_H_192_O_18_: C, 77.49; H, 9.05; found: C, 77.77; H, 9.09.

## Supporting Information

File 1 Characterization instruments and methods. ^1^H NMR spectra and ^13^C NMR spectra for the monomers and trimers.
