# Supplementary material for: Triphenylene discotic liquid crystal trimers synthesized by Co2(CO)8-catalyzed terminal alkyne [2 + 2 + 2] cycloaddition
Source: Beilstein J Org Chem. 2013 Dec 11;9:2852–61. doi: 10.3762/bjoc.9.321 (PMC3869258; doi:10.3762/bjoc.9.321)
Supplement: File 1 — Characterization instruments and methods. 1H NMR spectra and 13C NMR spectra for the monomers and trimers. [file Beilstein_J_Org_Chem-09-2852-s001.pdf]

Supporting Information

for

Triphenylene discotic liquid crystal trimers synthesized by $Co_2(CO)_8$-catalyzed terminal alkyne [2 + 2 + 2] cycloaddition

Bin Han[1], Ping Hu[1], Bi-Qin Wang[1], Carl Redshaw[1,2], Ke-Qing Zhao*[1]

Address:

[1]College of Chemistry and Materials Science, Sichuan Normal University, Chengdu 610066, China, and

[2]Department of Chemistry, University of Hull, Hull, HU6 7RX, UK

E-mail:

Ke-Qing Zhao - kqzhao@sicnu.edu.cn,

* Corresponding author

Characterization instruments and methods. 1H NMR spectra and 13C NMR spectra for the monomers and trimers.

Table of contents

Characterization instruments and conditions 2

^{1}H and ^{13}C NMR spectra of monomers **2** and **3a** – **c** 3-6

^{1}H and ^{13}C NMR spectra of trimers **4** and **5a** - **c** 7-10

Characterization instruments and conditions

^{1}H NMR (600 MHz) spectra were collected on a Bruker-Advance-600 spectrometer, and ^{1}H NMR (400 MHz) spectra were collected on a Varian UNITY INOVA-400 (400 MHz). For NMR characterization, $CDCl_3$ was used as the solvent and TMS as the internal standard. Elemental analysis was conducted on a CARLO ERBA 1106 machine. Phase transitions and enthalpy changes were measured using differential scanning calorimetry (DSC) on a TA-DSC Q100 instrument with heating and cooling rates of 10 K / min under an N_2 atmosphere. Liquid crystal textures were observed on an OLYMPUS BX41 polarizing optical microscope (POM) installed with a HCS302-GXY heating plate and a INSTEC STC200 temperature controller. 1D WAXD temperature dependant powder experiments were performed on a Rigaku Smartlab (3) X-Ray diffractometer equipped with a TCU 110 temperature control unit. The sample temperature was controlled within ± 1 K. The X-ray sources (Cu Kα, λ=0.154 nm) were provided by 40 kW ceramic tubes and with scan rate of 10 K/min.

NMR spectra for monomers 2 and 3a – c

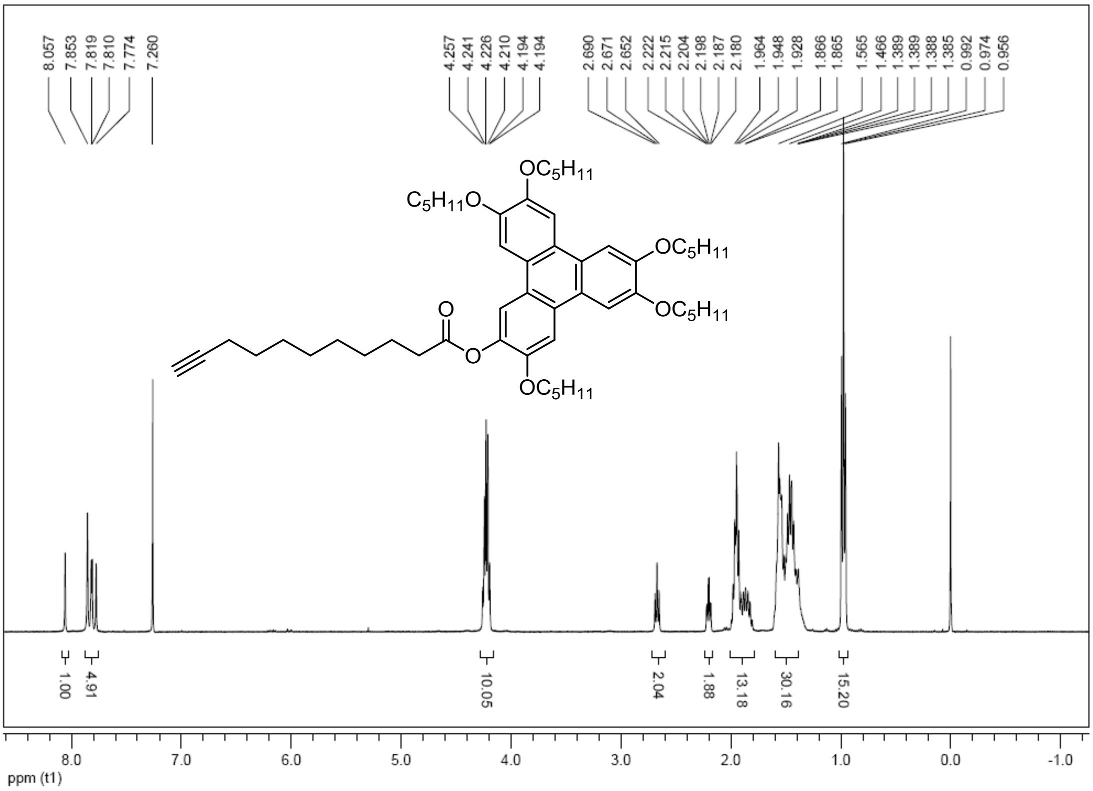

Figure S1: ^{1}H NMR ($CDCl_3$, 400 MHz) of monomer **2**.

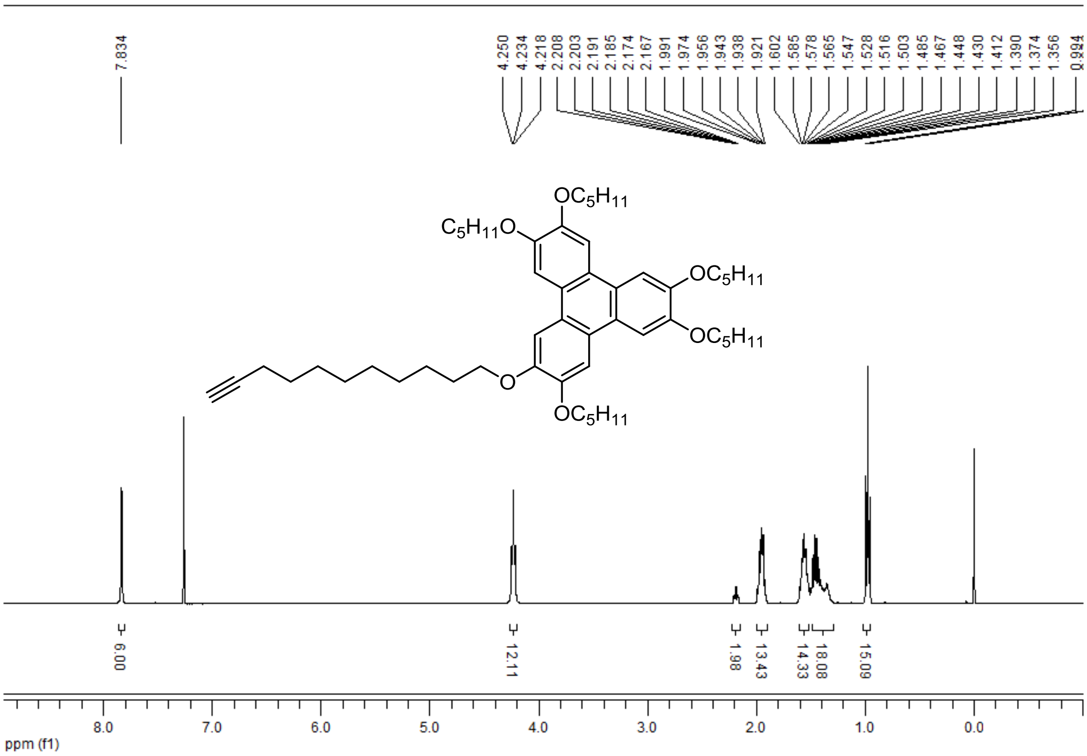

Figure S2: ^{1}H NMR ($CDCl_3$, 400 MHz) of monomer **3a**.

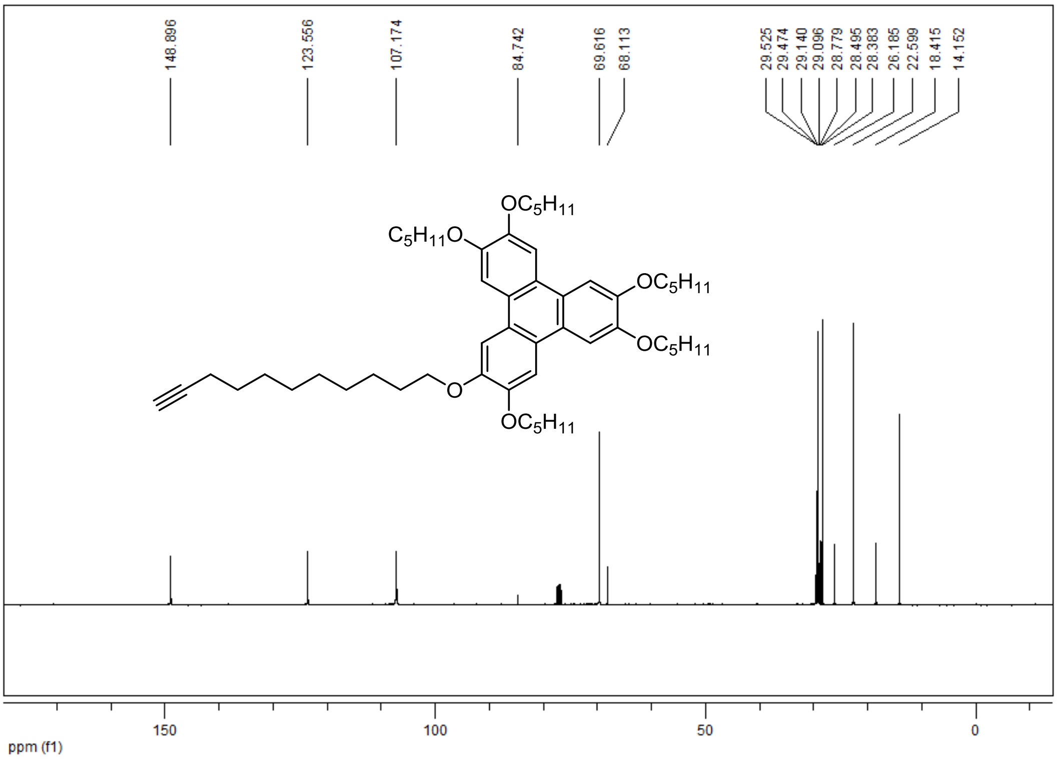

Figure S3: ^{13}C NMR ($CDCl_3$, 100 MHz) of monomer **3a**.

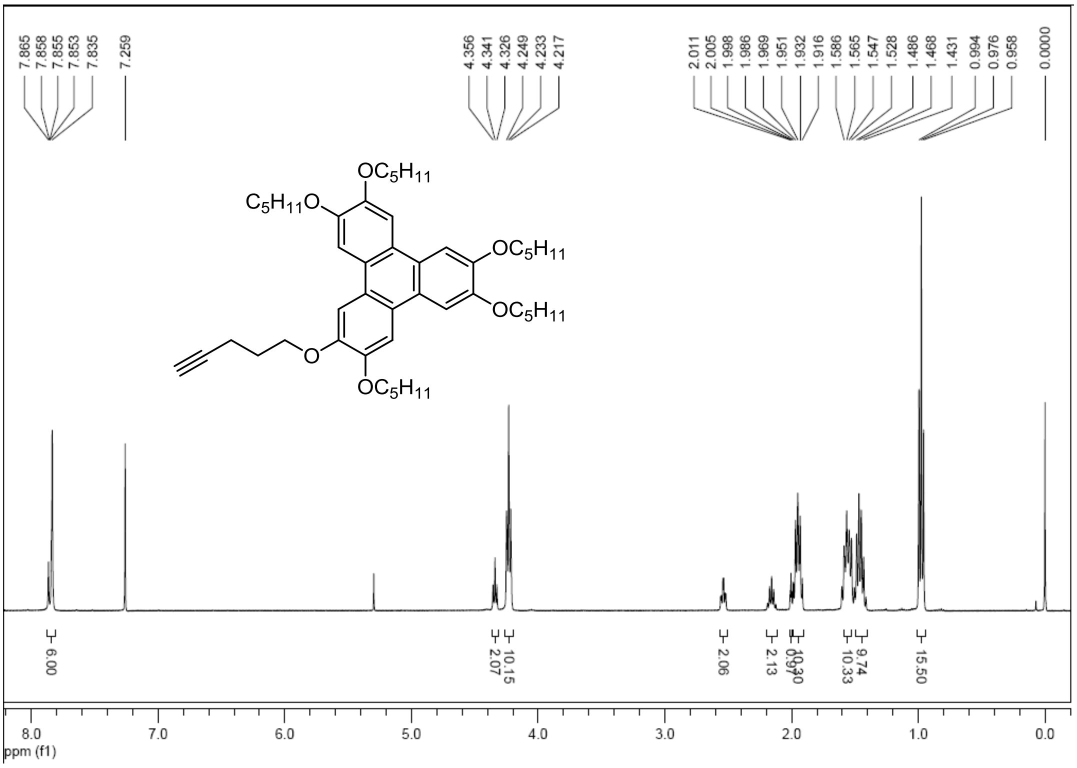

Figure S4: ^{1}H NMR ($CDCl_3$, 400 MHz) of monomer **3b**.

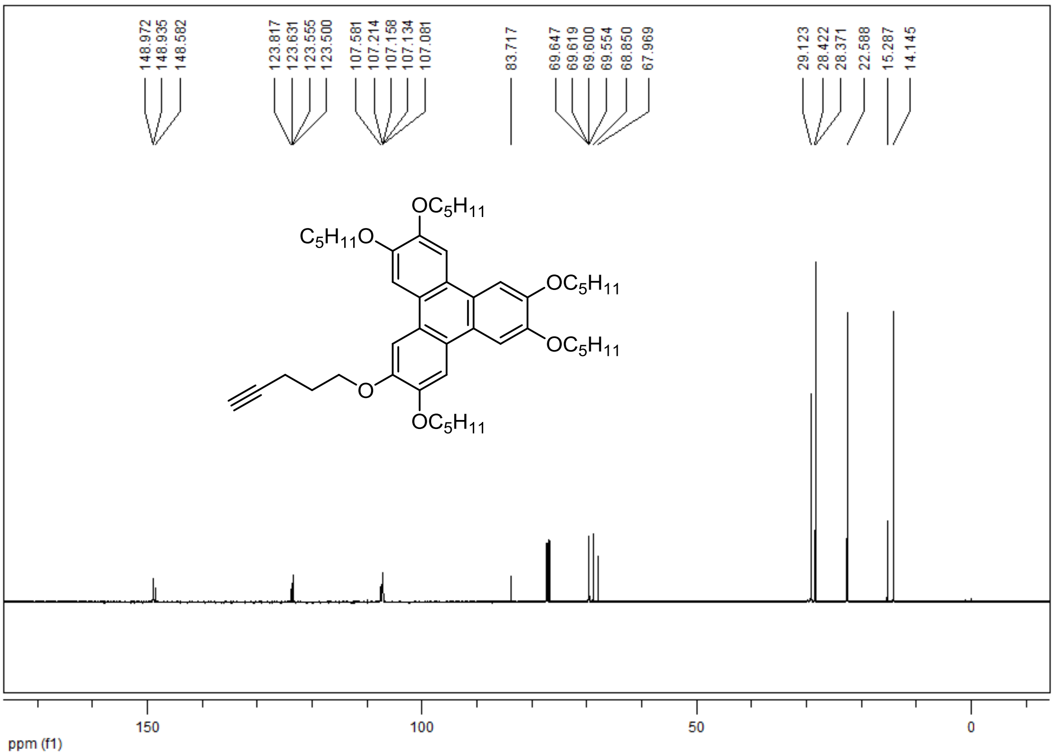

Figure S5: ^{13}C NMR ($CDCl_3$, 100 MHz) of monomer **3b.**

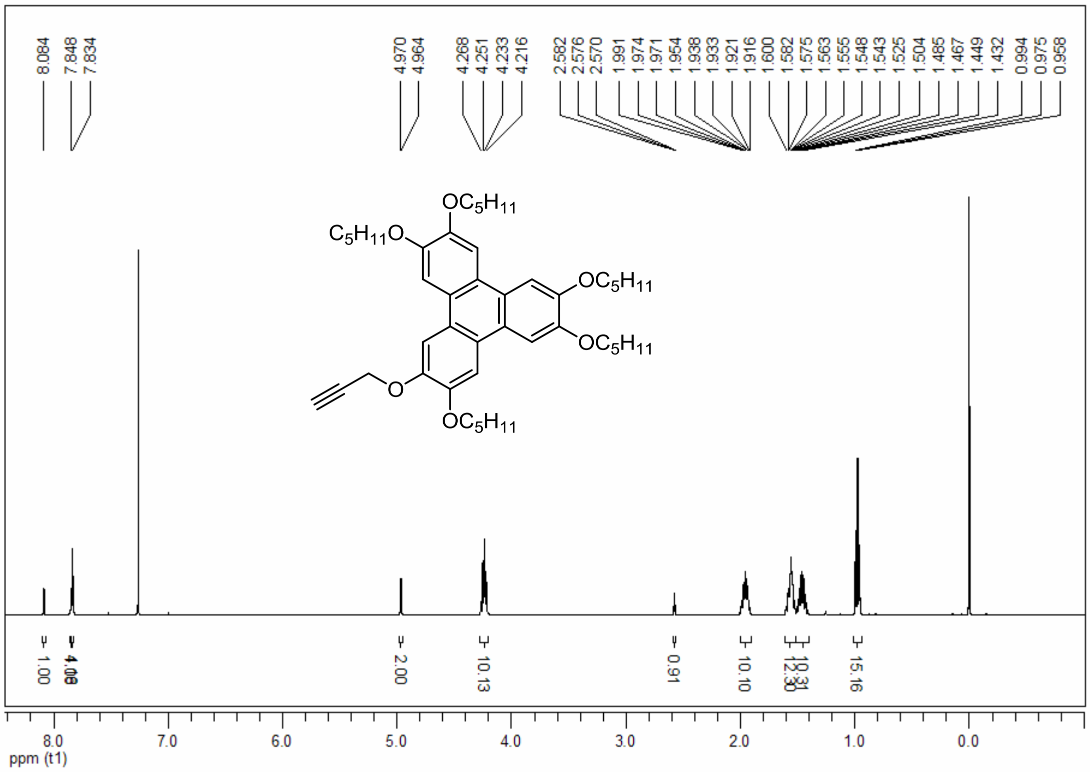

Figure S6: ^{1}H NMR ($CDCl_3$, 400 MHz) of monomer **3c**.

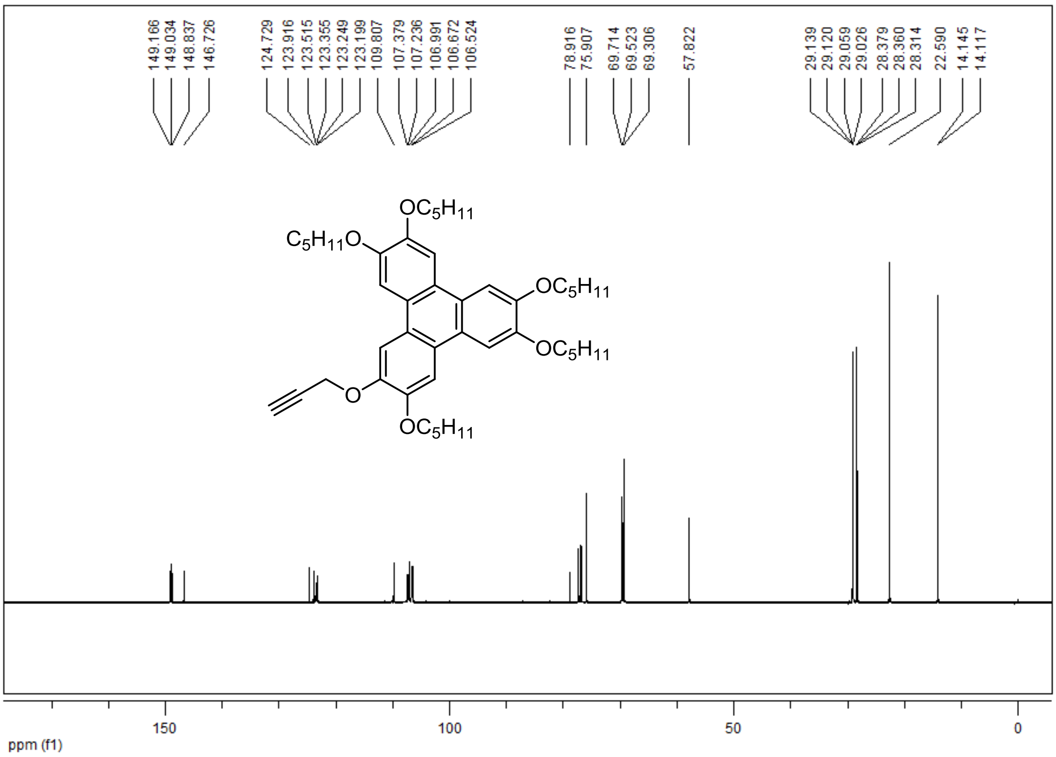

Figure S7: ^{13}C NMR ($CDCl_3$, 100 MHz) of monomer **3c.**

NMR spectra for trimers 4 and 5a – c

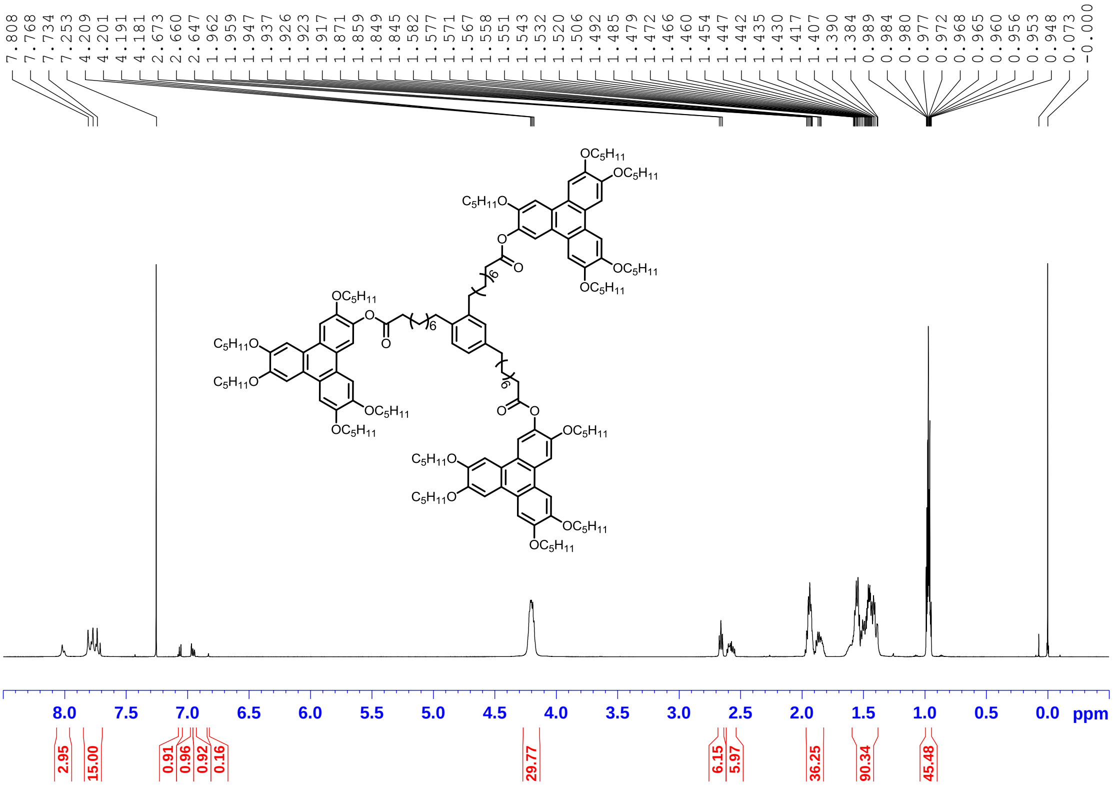

Figure S8: ^{1}H NMR ($CDCl_3$, 600 MHz) of trimer **4**.

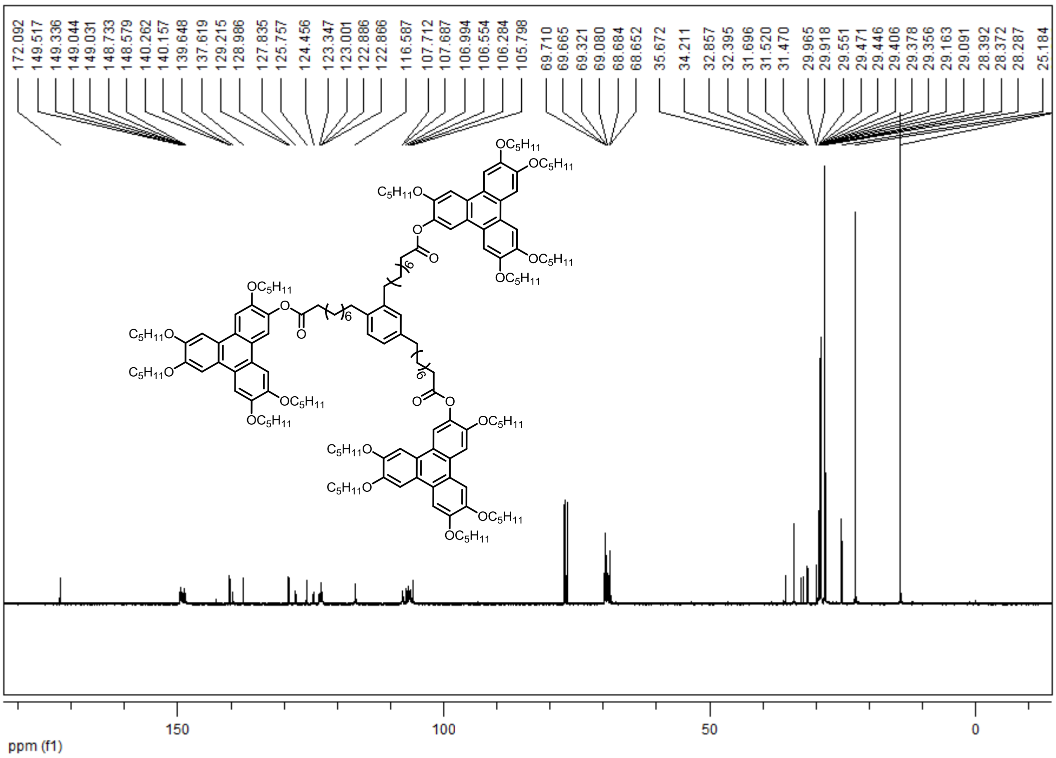

Figure S9: ^{13}C NMR ($CDCl_3$, 100 MHz) of trimer **4.**

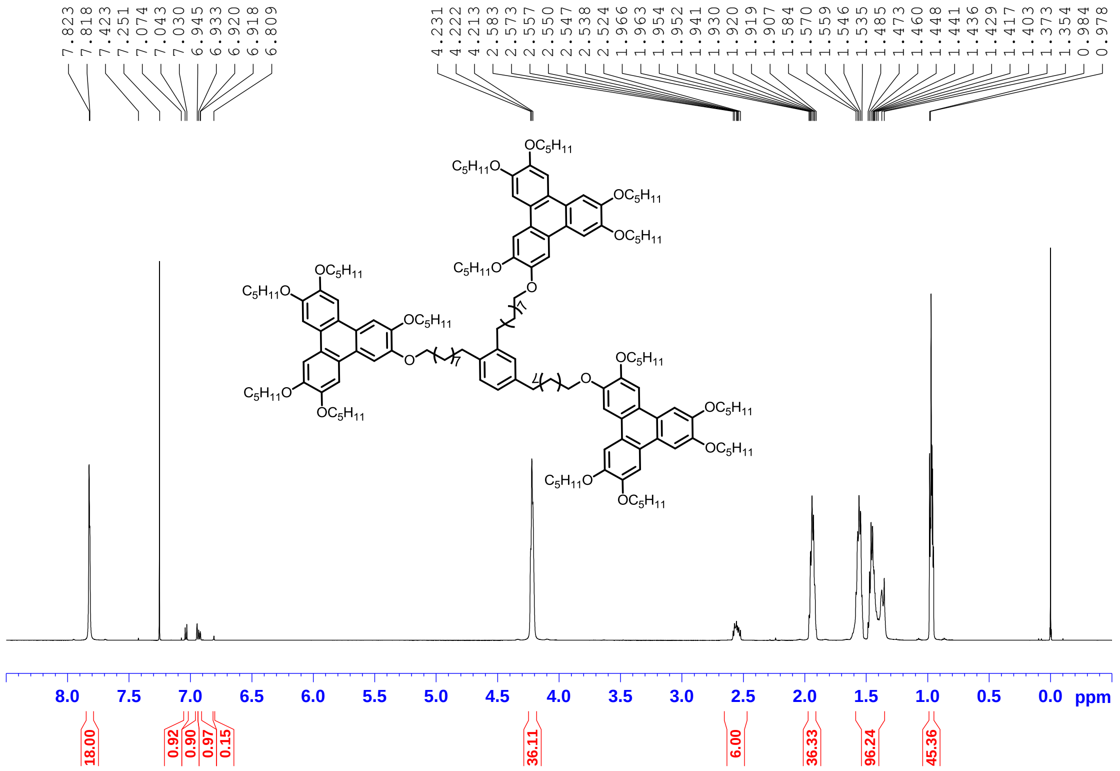

Figure S10: ^{1}H NMR ($CDCl_3$, 600 MHz) of trimer **5a**.

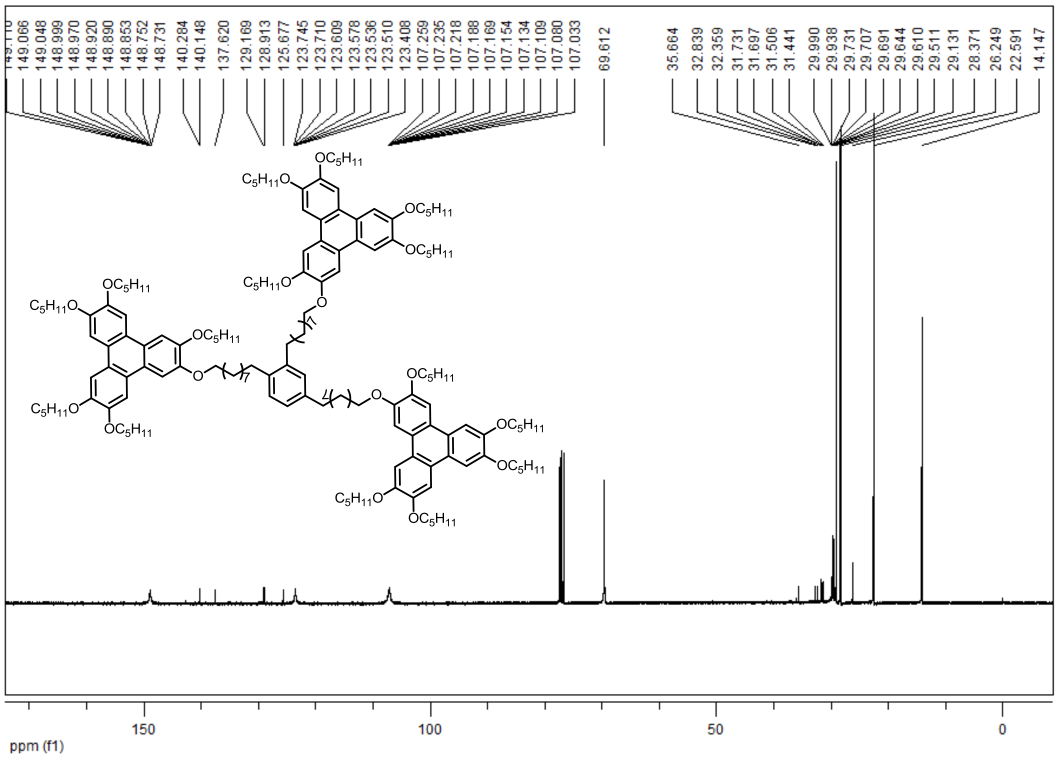

Figure S11: ^{13}C NMR ($CDCl_3$, 100 MHz) of trimer **5a.**

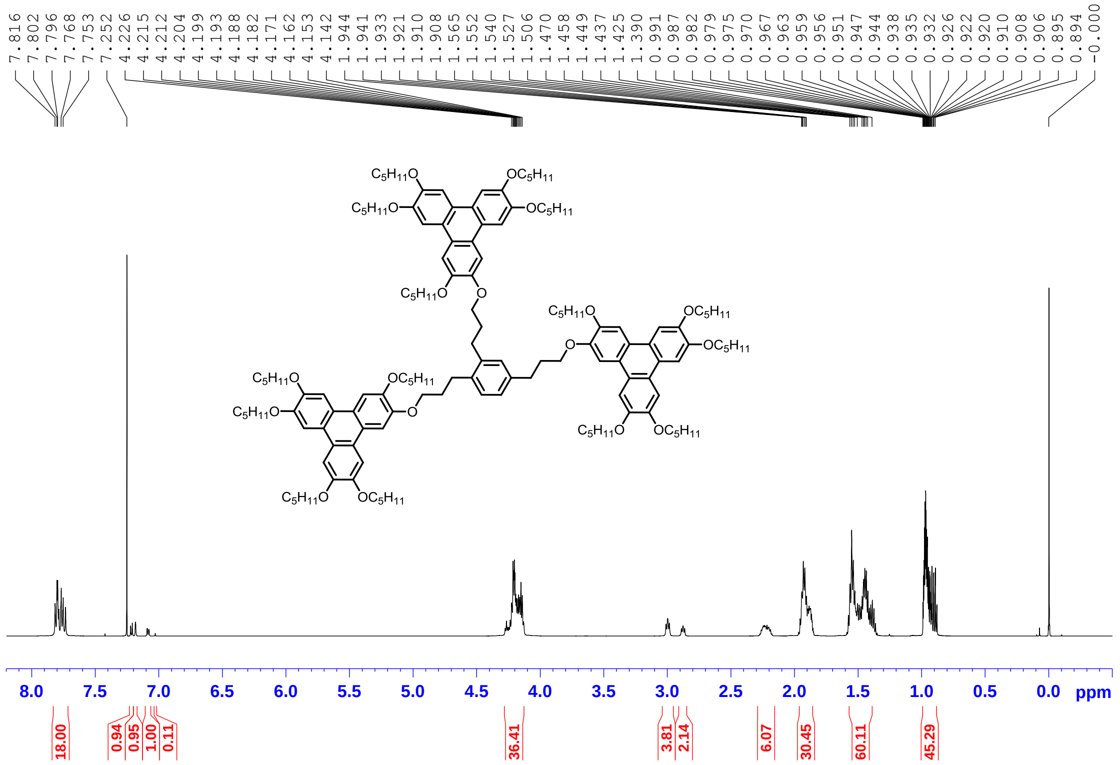

Figure S12: ^{1}H NMR ($CDCl_3$, 600 MHz) of trimer **5b**.

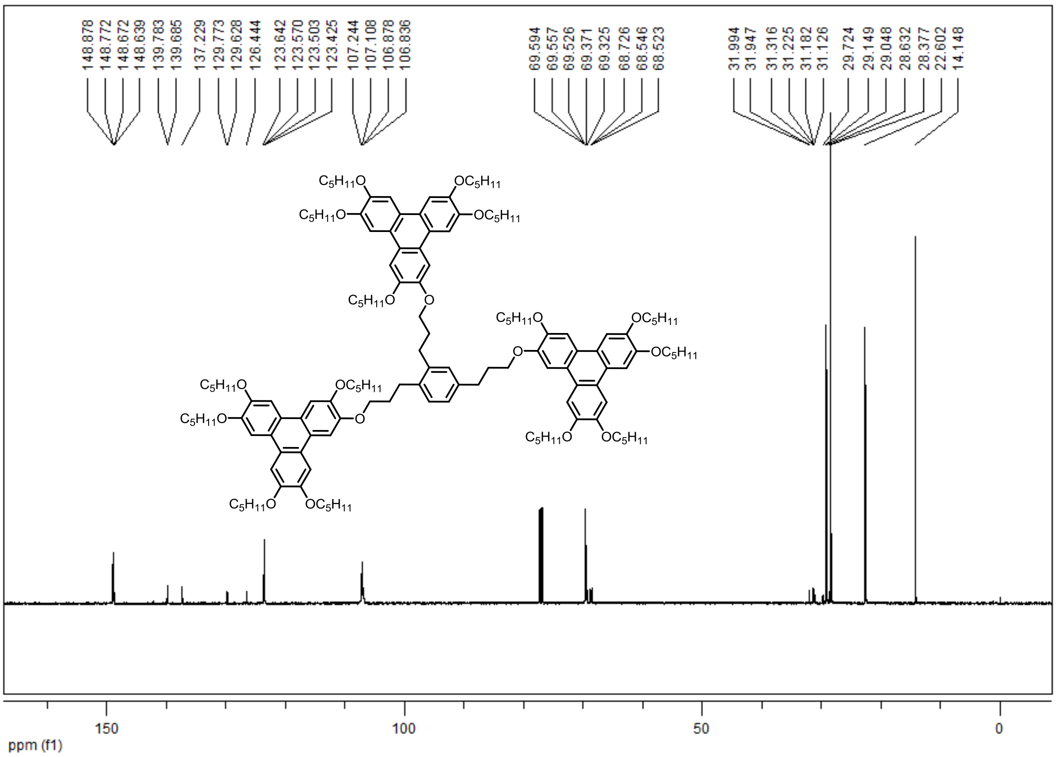

Figure S13: ^{13}C NMR ($CDCl_3$, 100 MHz) of trimer **5b.**

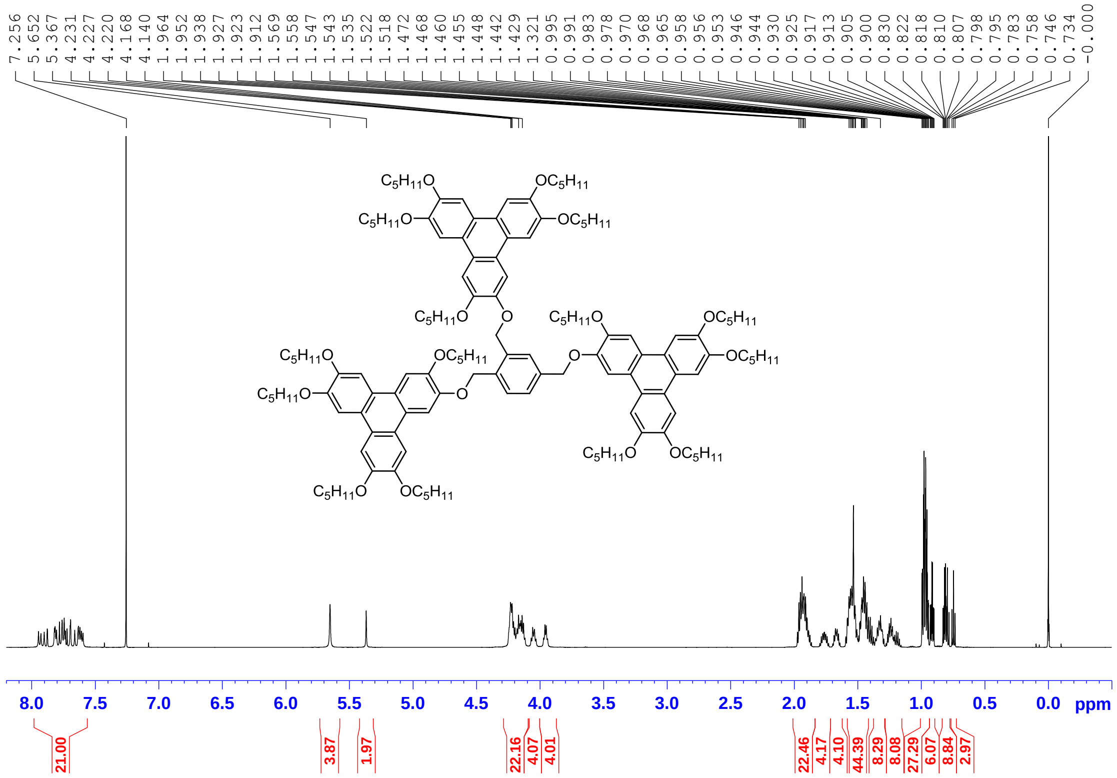

Figure S14: ^{1}H NMR ($CDCl_3$, 600 MHz) of trimer **5c**.

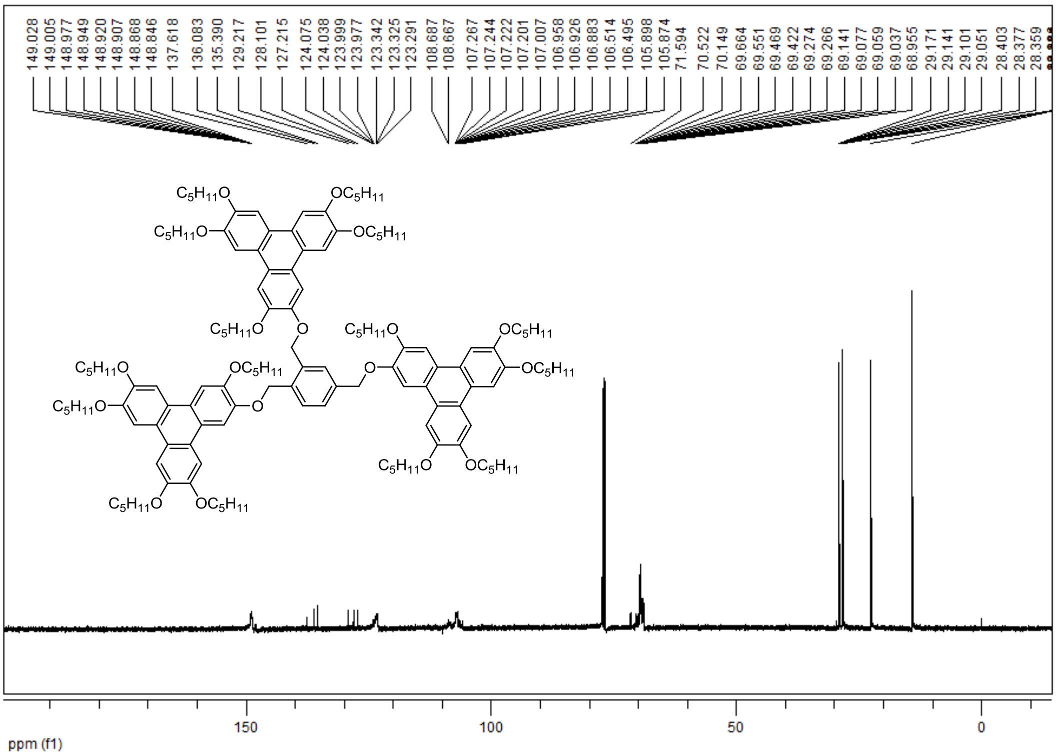

Figure S15: ^{13}C NMR ($CDCl_3$, 100 MHz) of trimer **5c.**
